# Long-Term Injury Patterns and Trends in Elite Professional Football: A Nine-Season Retrospective Analysis of Prospectively Recorded Data

**DOI:** 10.3390/life16091517

**Published:** 2026-09-11

**Authors:** Ante Bandalović, Šime Veršić, Jakša Škomrlj, Tomislav Pranjić, Arian Skoki, Marin Marinović, Ante Turić, Marko Kordić, Bruno Lukšić

**Affiliations:** 1 Division of Orthopaedics and Traumatology, Department of Surgery, University Hospital of Split, 21000 Split, Croatia; ante.bandalovic@hajduk.hr (A.B.); ante.turic@hajduk.hr (A.T.); marko.kordic@hajduk.hr (M.K.); brluksic@kbsplit.hr (B.L.); 2 Hrvatski Nogometni Klub Hajduk Split, 21000 Split, Croatia; 3 Faculty of Kinesiology, University of Split, 21000 Split, Croatia; skomrljj@gmail.com (J.Š.); tomislav.pranjic@kifst.eu (T.P.); 4 Football Club Noah, Yerevan 0023, Armenia; 5 Department of Computer Engineering, Faculty of Engineering, University of Rijeka, Vukovarska 58, 51000 Rijeka, Croatia; arian@gotofizio.com; 6 Department of Orthopedics and Traumatology, Surgery Clinic, KBC Rijeka, 51000 Rijeka, Croatia; marin.marinovic2@gmail.com

**Keywords:** professional football, injury epidemiology, injury surveillance, time-loss injury, injury burden, muscle injury, longitudinal study, Joinpoint regression

## Abstract

Long-term injury surveillance in professional football is essential for understanding temporal changes in injury patterns. However, prospective studies spanning multiple consecutive seasons within the same professional club remain scarce. This study aimed to investigate injury epidemiology, temporal trends, and injury characteristics over nine consecutive seasons in an elite Croatian professional football club. This retrospective longitudinal study over nine competitive seasons (2015/16–2023/24) included 167 male players participating in the professional first-team environment from a single professional football club, contributing a total of 382 player-seasons across nine consecutive seasons. A total of 749 time-loss injuries were recorded according to the FIFA Medical Assessment and Research Centre (F-MARC) consensus recommendations. Temporal trends were evaluated using Joinpoint regression, while injury characteristics, anatomical distribution, tissue involvement, and injury burden were analysed descriptively. Overall injury incidence averaged 12.66 injuries per 1000 h of exposure (95% CI: 11.77–13.60), with substantially higher incidence during matches (50.13 injuries/1000 h) than training (7.97 injuries/1000 h). Joinpoint regression identified a non-significant annual decline in injury incidence before the COVID-19 season (2019/2020) (APC = −9.66%), followed by a non-significant increase thereafter (APC = 4.88%). At least one injury was recorded in 295 player-seasons, corresponding to 77.2% of all eligible player-seasons. More than 80% of all injuries involved the lower extremities, while muscle injuries accounted for almost half of all recorded injuries. Functional muscle disorders were the most frequent injury type, whereas meniscal and articular cartilage injuries were associated with the highest mean time loss per injury. The predominance of lower-limb muscle injuries, together with the substantial burden associated with less frequent intra-articular knee injuries, highlights key priorities for injury prevention in professional football. Long-term surveillance within the same club provides valuable insights into evolving injury patterns and supports the development of targeted strategies to maximise player availability.

## 1. Introduction

Over the past two decades, the physical demands of elite football have increased significantly because of higher match intensity, greater tactical complexity, and congested competition schedules [1,2]. Modern players are required to perform repeated high-intensity actions while maintaining technical and tactical performance throughout the match [3,4]. These activities impose considerable mechanical and physiological stress on the musculoskeletal system. Elite football players are also exposed to substantial cumulative workloads through regular training sessions, travel, and repeated competitive fixtures across prolonged seasons [5,6]. The interaction between all these factors creates a complex environment in which injury risk is continuously influenced by both acute and chronic stressors [7]. Systematic injury surveillance is essential for understanding the frequency and consequences of injuries in professional football and for informing targeted risk-reduction strategies. Injury incidence describes the number of injuries relative to player exposure, whereas injury severity reflects the duration of absence associated with an individual injury. Injury burden integrates these dimensions by expressing the total number of days lost per 1000 h of exposure, thereby providing a broader estimate of the impact of injuries on player availability. Therefore, injury prevention and maintaining player availability have become a major priority for coaching and medical staff. Despite continuous advances in sports science, medicine, and athlete monitoring, injuries remain an inevitable part of elite football with direct consequences on individual performance, team success, clubs’ finances, and long-term player development [8,9].

Injuries in professional football have become one of the most extensively investigated topics in sports medicine over the past two decades [10,11,12]. Reported injury incidence in football varies considerably across studies, with values ranging from approximately 1.6 to 19.2 injuries per 1000 h of exposure. This broad range should be interpreted in light of differences in competitive level, age and sex of the studied populations, injury definitions, surveillance procedures, and the type of exposure examined [8,13,14]. In studies specifically involving elite male professional players and applying a time-loss injury definition, overall injury incidence is more commonly reported to be approximately 6–10 injuries per 1000 h of combined training and match exposure [8].

Recent longitudinal evidence has suggested that while overall injury incidence in elite football has gradually decreased over time, the proportion and burden of muscle injuries, especially hamstrings, have continued to increase [8,12]. This indicates that soft tissue injuries remain difficult to control despite advances in training methodology and injury prevention protocols. The UEFA Elite Club Injury Study, the largest and longest-running prospective surveillance programme in professional football, shows that injury incidence is consistently higher during matches than training, with players sustaining roughly two time-loss injuries per season [8,10]. Regarding injury patterns, findings between the studies are generally consistent with muscle injuries accounting for the largest proportion, followed by ligament injuries, while the thigh represents the most frequently affected anatomical region [12]. Also, recurrent injuries continue to represent an important issue and clinical challenge, as they are typically associated with longer recovery periods and greater time-loss than index injuries [15]. In addition to being a direct health and medical problem for the individual, injuries affect the team’s success and represent a financial burden. Studies have reported that greater player availability is associated with improved team success, as teams with higher injury incidence achieve a lower average number of points per game [16]. Additionally, injuries represent a significant cost, both direct in terms of expenses for treatment and rehabilitation, and indirect, as teams spend money on players who are not performing [17]. Continuous injury surveillance enables clubs to identify changing injury patterns, evaluate preventive strategies, and adapt medical and training practices to the evolving demands of professional football.

Although many studies have investigated the epidemiology of injuries in professional football over the past two decades, there are certain gaps in knowledge. Also, given the constant increase in playing intensity and technical-tactical trends, it is necessary to continuously monitor any changes in injury patterns. Most published studies have evaluated injury incidence over a single season or a limited number of competitive seasons, providing valuable single-season prospective information but offering only limited insight into how injury patterns change [13,18,19]. The UEFA Elite Club Injury Study represents a valuable source of information and the longest-running prospective surveillance programme in professional football [10,12]. However, its multicenter design primarily describes overall trends across participating clubs rather than the long-term injury profile of an individual club operating within a consistent sporting and medical environment. Additionally, it includes the most successful and financially strongest clubs, predominantly from Western and Northern Europe, which may limit the generalizability of the findings, given that they are the most advanced systems with the most valuable players. To the best of our knowledge, no study has comprehensively described injury epidemiology across multiple consecutive seasons in Croatian elite professional football and even in the top-tier leagues in the region. A previous study based on eight seasons from the same club (2016/17–2023/24) investigated changes in overall and muscle-injury incidence during the two- and four-week periods following coaching transitions [11]. The present study uses the same underlying club database, supplemented by data from the 2015/16 season, but addresses a distinct research question. Specifically, it provides a comprehensive nine-season epidemiological analysis of injury location, type, mechanism, severity, time loss, burden, and long-term temporal trends, none of which were the primary focus of the previous study. In addition, although several studies have investigated temporal changes in specific injury categories, such as hamstring or groin injuries, relatively few have examined overall injury trends alongside injury characteristics, anatomical distribution, tissue type, and injury burden within the same cohort [20,21]. Long-term surveillance within a single professional club enables the evaluation of injury trends in a relatively consistent cohort and within a relatively stable medical surveillance system, characterised by continuity of the medical personnel and consistent injury-recording and diagnostic procedures. Such evidence may contribute to a better understanding of injury epidemiology and inform future injury prevention strategies in elite professional football.

Therefore, the main aim of this study was to investigate the epidemiology of time-loss injuries over nine consecutive seasons in an elite Croatian professional football club, with a particular focus on injury incidence, temporal trends, injury characteristics, anatomical distribution, tissue type, and injury burden. We hypothesised that injury incidence would demonstrate a non-linear temporal pattern across the nine competitive seasons, with a change in trend around the COVID-19-interrupted 2019/20 season and an increase following the resumption of regular competition. However, any temporal correspondence with the pandemic was considered exploratory and was not interpreted as evidence of a causal effect.

## 2. Materials and Methods

### 2.1. Study Design

This study was a retrospective longitudinal analysis of prospectively and routinely recorded medical and exposure data from the first team of a single professional football club competing in the highest tier of Croatian football across nine consecutive seasons (2015/2016–2023/2024). Academy players contributed injury and exposure data only during their participation in first-team activities. Injury data were prospectively collected and stored in the club’s medical database, where all injuries were diagnosed and documented by the club’s medical staff, including team physicians and physiotherapists, following routine clinical practice. The Faculty of Kinesiology’s Ethics Committee gave its approval for this study, which was carried out in compliance with the Declaration of Helsinki (Ethical Approval Number: 2181-205-02-05-23-0007). The approval included the retrospective use of medical and exposure data routinely recorded by the club before the date of ethical approval. During their contractual relationship with the club, players had agreed that data obtained through injury surveillance and performance testing could be used in anonymised form for analytical purposes. Accordingly, historical records of players who had left the club before the ethical approval were included without re-contacting them, under the conditions approved by the Ethics Committee. All historical data were anonymised before analysis and reported exclusively at the group level. Following ethical approval, adult participants provided written informed consent for the research use of their data. For participants younger than 18 years, written consent was obtained from a parent or legal guardian, with assent from the player.

### 2.2. Participants

A total of 167 unique players (382 season-players) were included across the nine competitive seasons, during which 749 time-loss injuries were recorded. The study population comprised players participating in the professional first-team environment. This included first-team players and academy players who were temporarily integrated into first-team training or competition. Academy players contributed data only for the periods during which they participated in first-team activities. Players were included if they remained with the club for at least half of a competitive season. Consequently, players who permanently left the club during the winter transfer window were excluded from the study in the corresponding season. Players who participated in more than one season contributed one player-season for each season of participation. However, player-seasons were not treated as independent observations in the temporal trend analysis, which was based on nine aggregated season-specific injury incidence rates and their corresponding exposure values. Each observation period covered the complete annual training and competition cycle, including the summer pre-season, the first half of the competitive season, the winter pre-season, and the second half of the competitive season. Across all nine seasons, the team competed in UEFA club competitions during July and August (2–6 matches per season), followed by the Croatian First Football League (36 matches) and the Croatian Cup (3–6 matches) during the remainder of the season. The only exception was the 2019/2020 season, when the COVID-19 pandemic resulted in a four-week suspension of all team training during March and April 2020. This was followed by a three-week period in which players trained individually or in small groups before regular team training resumed.

### 2.3. Variables

All injuries were prospectively recorded by the club’s medical staff daily and documented in the club’s medical database, in accordance with the recommendations of the FIFA Medical Assessment and Research Centre (F-MARC) consensus statement [22]. This framework was retained throughout the observation period to ensure consistent classification across all nine seasons. Following the publication of the more recent IOC consensus statement and its football-specific extension, the historical dataset was reviewed for compatibility with the updated recommendations. Core variables, including time-loss injury, exposure, injury location, type, mechanism, severity, and burden, were harmonised with the newer reporting recommendations where the available data permitted. Variables that had not been prospectively recorded during the earlier seasons were not retrospectively inferred or reclassified. An injury was defined as any physical complaint sustained during football activities that resulted in a player being unable to participate fully in subsequent training sessions or matches. All injuries were assessed and ultimately diagnosed by the club physicians, who remained unchanged throughout the nine-season observation period. Diagnoses were based on clinical examination and supported by diagnostic imaging when clinically indicated. Ultrasound examinations were performed using the club’s ultrasound equipment, while magnetic resonance imaging was obtained, when necessary, through collaborating clinical institutions.

Injuries were categorised as contusions, functional muscle disorders—muscle cramps and delayed onset muscle soreness (DOMS), tendinosis, ligament injuries (sprains/dislocations), structural muscle disorders—muscle ruptures, other bone injuries, fractures, meniscal or cartilage lesions, bursitis, tendon ruptures, concussions, abrasions, and lacerations. For each injury, additional information was recorded, including the number of days lost from team training and match participation, injury character (trauma or overuse), injury setting (training or match), injury mechanism (contact or non-contact), and whether the injury represented a recurrence. A recurrent injury was defined according to the consensus statement as an injury of the same type affecting the same anatomical location after the player had returned to full participation. Missing injury-classification data were not imputed. Injuries with an unavailable classification were retained in the calculations of overall injury incidence, total days lost, and injury burden and were reported as “unknown” for the relevant descriptive variable. They were excluded only from analyses requiring that specific classification.

Injury severity was determined according to the duration of absence from full training and match participation and was reported as the total number of days lost (days out). Injury distribution according to the affected body region and tissue type was also analysed. To provide a comprehensive overview of the impact of different injury types, injury incidence, and the mean number of days lost were presented simultaneously in a bubble plot, with bubble size representing the total number of injuries.

Football exposure was quantified separately for training and matches and expressed in hours. Match exposure was calculated from the actual playing time of each participating player, including both starters and substitutes. Extra time was included when applicable, while exposure for players who were substituted or dismissed ended at the recorded minute of substitution or dismissal. Training exposure was calculated according to each player’s actual duration of participation in regular team training sessions. Where a player participated in only part of a team session, only the corresponding minutes were included. Rehabilitation sessions and individual training performed outside regular team training were excluded. Injury incidence was then calculated as the number of injuries per 1000 h of exposure and was calculated separately for training, match, and overall exposure. For academy players integrated into the first team, exposure included only training sessions completed with the first team and first-team matches in which they participated. Exposure accumulated during B-team or U19 training sessions and matches was excluded. Correspondingly, only injuries sustained during first-team training or match participation were included, whereas injuries occurring during B-team or U19 activities were not recorded as study outcomes. In addition to injury incidence, the proportion of injured players was calculated for each season and expressed as the percentage of players sustaining at least one injury relative to the total number of players included during that competitive season.

### 2.4. Statistics

Data were analysed using descriptive statistics. Continuous variables were summarised as arithmetic means, whereas categorical variables were reported as frequencies and percentages. Injury incidence was calculated as the number of injuries per 1000 h of exposure and is presented with corresponding 95% confidence intervals (95% CI). Differences in injury incidence were evaluated using incidence rate ratios (IRRs) with corresponding 95% confidence intervals.

Trends in injury incidence across the nine-season observation period were assessed using the Joinpoint Regression Programme (version 4.9.1.0; Statistical Research and Applications Branch, National Cancer Institute, Bethesda, MD, USA). Microsoft Excel 2019 (Microsoft Corp., Redmond, WA, USA) and GraphPad Prism (GraphPad Software 8.4.2., Boston, MA, USA) were used for data processing and graphical presentation. Statistical significance was set at *p* < 0.05.

## 3. Results

Injury incidence rates are presented in Table 1. Overall incidence ranged from 8.04 (95% CI: 5.79–10.86) in the 20/21 season to 17.93 (95%CI: 14.87–21.45) in the 16/17 season. Match injury incidence was consistently higher than training injury incidence across all seasons. The highest training injury incidence was in the 16/17 season, with 12.78 (95% CI: 10.07–15.99) injuries per 1000 h of training. The highest match incidence was reached in the 17/18 season, at 70.92 (95% CI: 53.43–92.31) injuries per 1000 match hours. In the endpoint comparison, injury incidence in the final season was 0.85 (95% CI: 0.62–1.16) times that observed in the first, indicating no statistically significant overall change in injury incidence over time.

**Figure 1:**
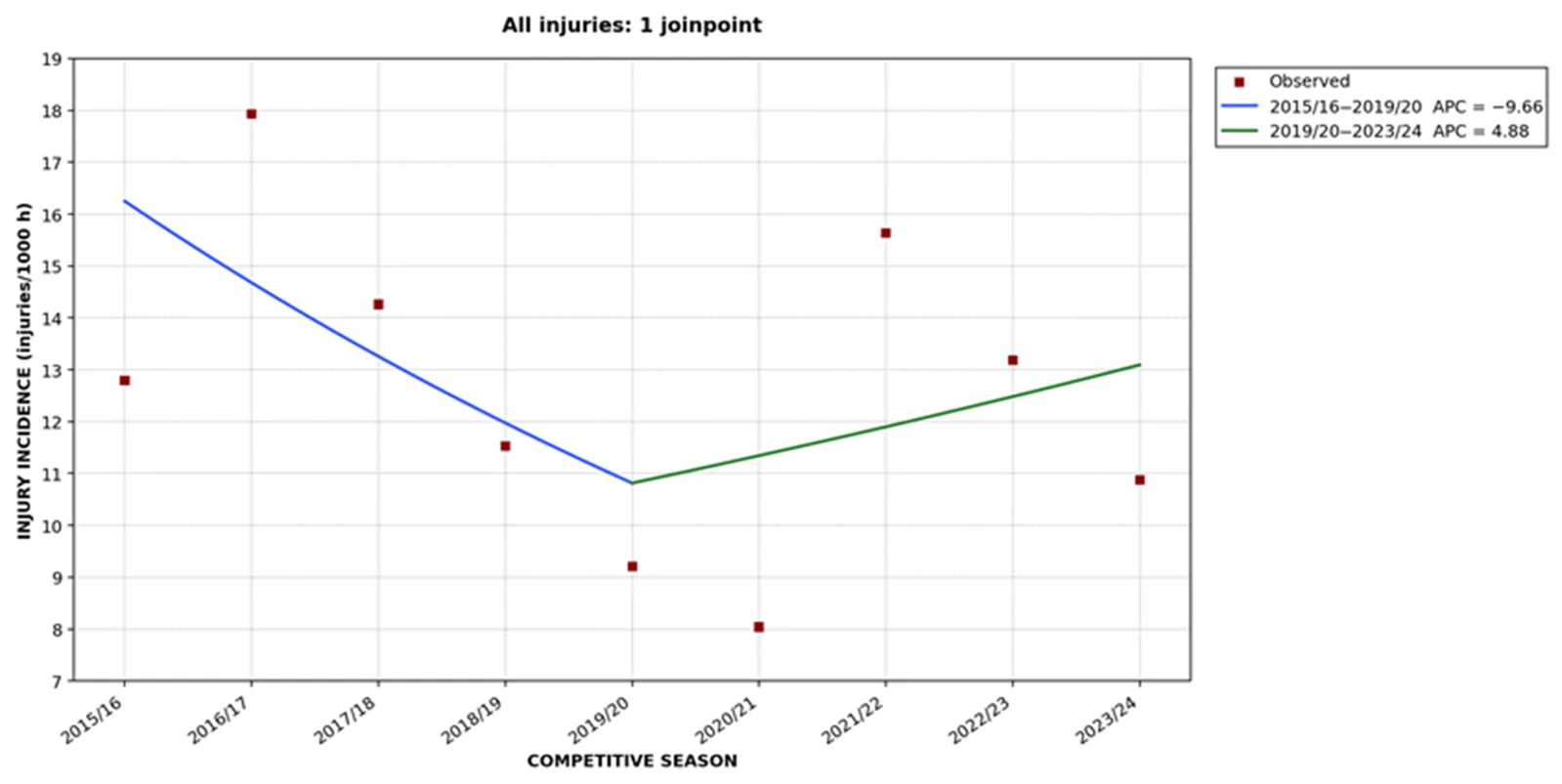
Joinpoint regression—injury trend through seasons.

The Joinpoint regression model identified one joinpoint during the study period (Figure 1). Injury incidence showed a non-significant estimated annual decrease from 2015/16 to 2019/20 (APC = −9.66%; 95% CI: −37.64 to 33.72; *p* = 0.238), followed by a non-significant estimated annual increase from 2019/20 to 2023/24 (APC = 4.88%; 95% CI: −32.60 to 58.96; *p* = 0.471). The overall trend across the nine seasons was also not statistically significant (AAPC = −2.66%; 95% CI: −12.02 to 6.34; *p* = 0.445).

A total of 749 injuries were recorded during the observed 9-season period as presented in the Table 2. The highest number of injuries was recorded in the 2016/17 season (n = 120), whereas the lowest was observed in 2020/21 (n = 42).

**Figure 2:**
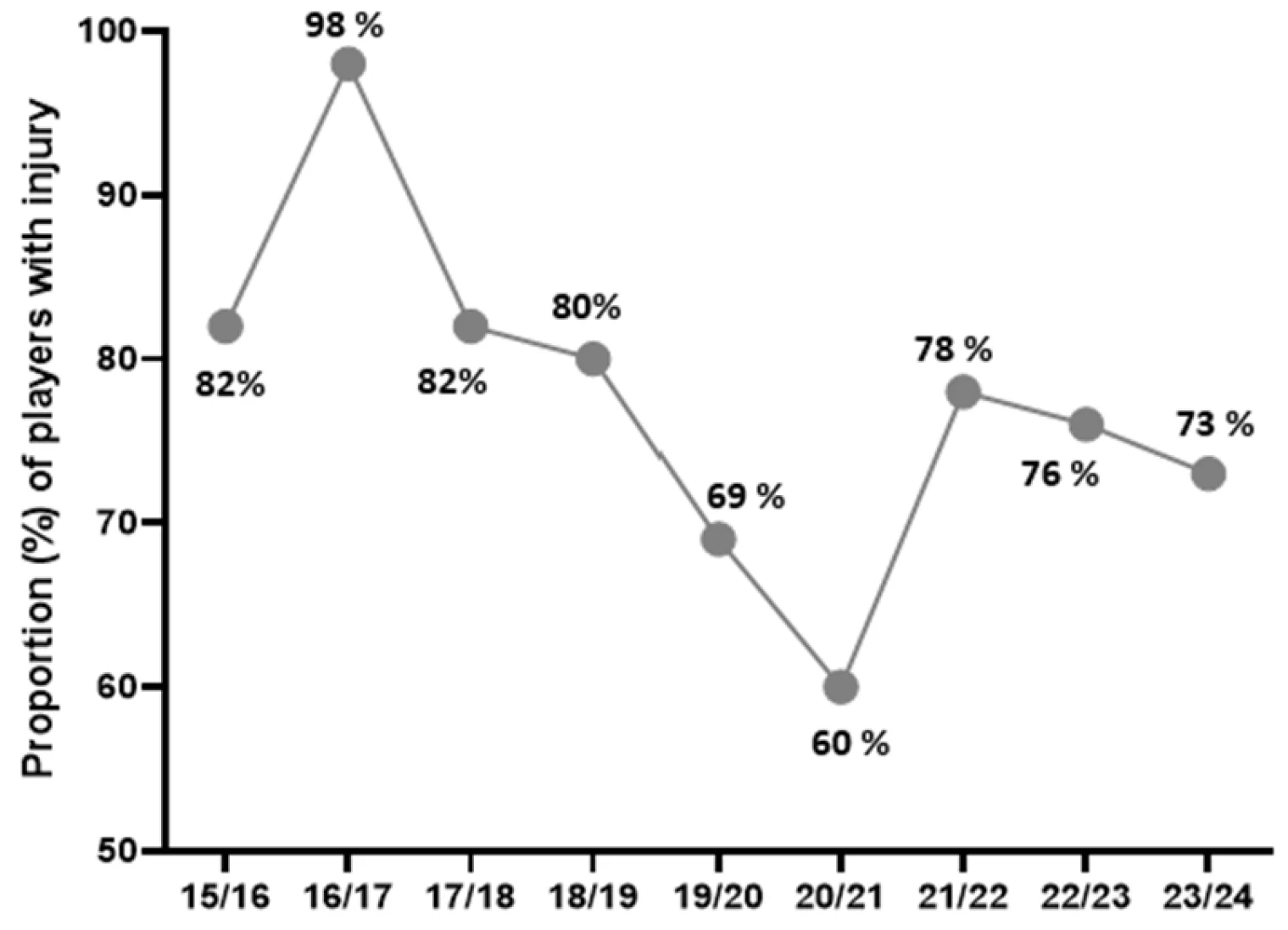
Proportion of injured players.

Figure 2 shows seasonal trends in the proportion of injured players, with a range from 60% in the 20/21 season to 98% in the 16/17 season.

Descriptive characteristics of injuries are presented in Table 3. Functional muscle disorders (muscle cramps/DOMS) were the most frequently recorded injury (n = 240), followed by ligament injuries (n = 128) and contusions (n = 118). Meniscal or cartilage lesions (48.7 days) and tendon ruptures (48.0 days) were associated with the longest mean absence from training and competition, whereas contusions (3.63 days) and lacerations (3.0 days) resulted in the shortest absence. Most ligament injuries, contusions, fractures, concussions, and tendon ruptures occurred as a consequence of trauma, whereas tendinosis and other bone injuries were predominantly classified as overuse injuries. Functional muscle disorders and tendinosis occurred more frequently during training, while others were more common during matches. The highest number of recurrent injuries was observed for functional muscle disorders (n = 91), followed by ligament injuries (n = 26), tendinosis (n = 24), and other bone injuries (n = 19).

Values within each classification category sum to the corresponding total number of injuries. “Unknown” indicates that the relevant information could not be established from the available medical record, whereas “not applicable” indicates that the classification was not relevant to the injury concerned. No missing classifications were imputed.

**Figure 3:**
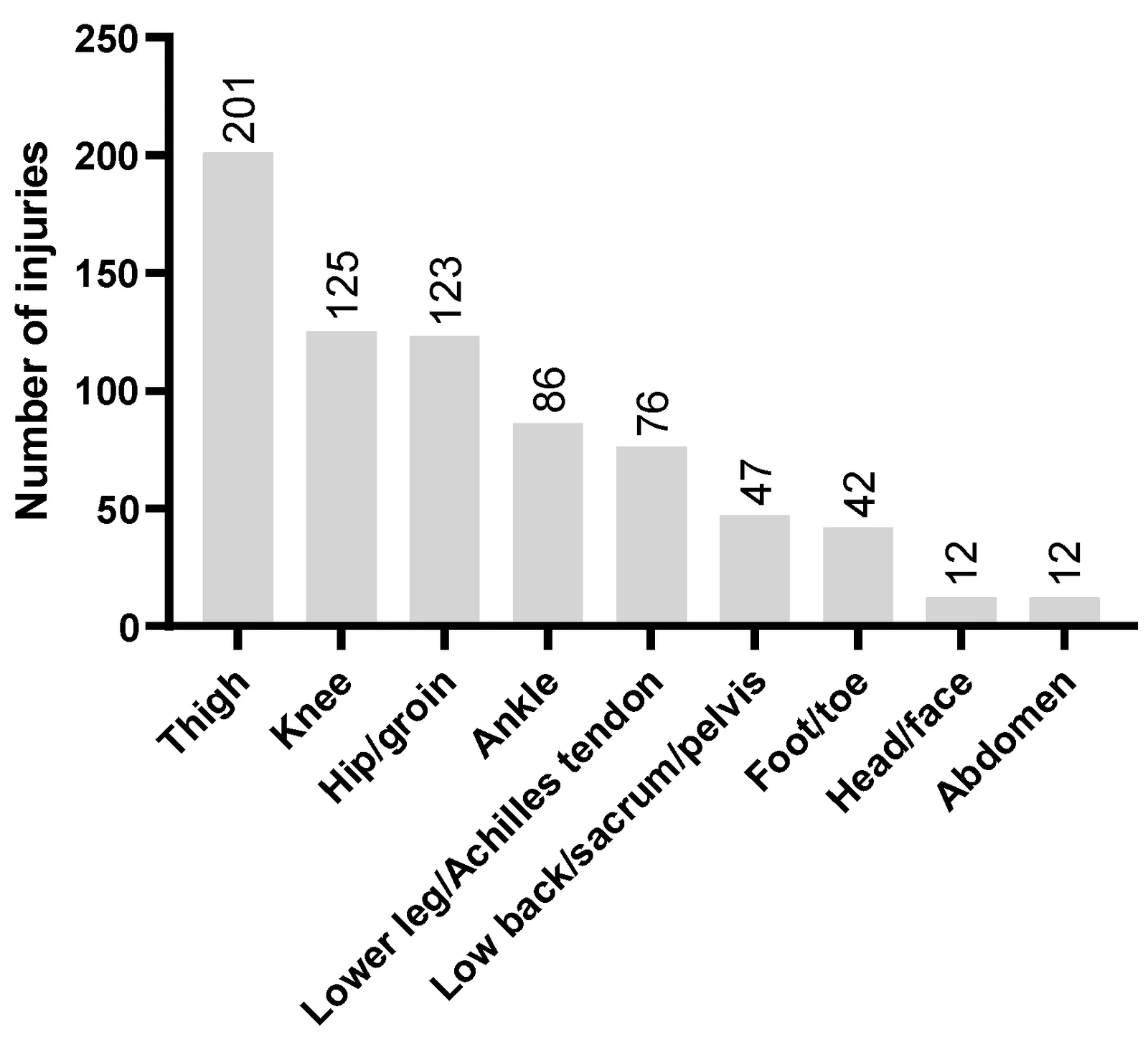
Injured body parts.

The distribution of injuries according to the affected body region is presented in Figure 3. Regions with a small number of injuries were omitted to improve visual clarity; therefore, the displayed values do not sum to the total of 749 injuries. The thigh was the most frequently injured body region (n = 201), followed by the knee (n = 125), hip/groin (n = 123), ankle (n = 86), and lower leg (n = 76). Together, these five anatomical regions accounted for approximately 80% of all recorded injuries.

**Figure 4:**
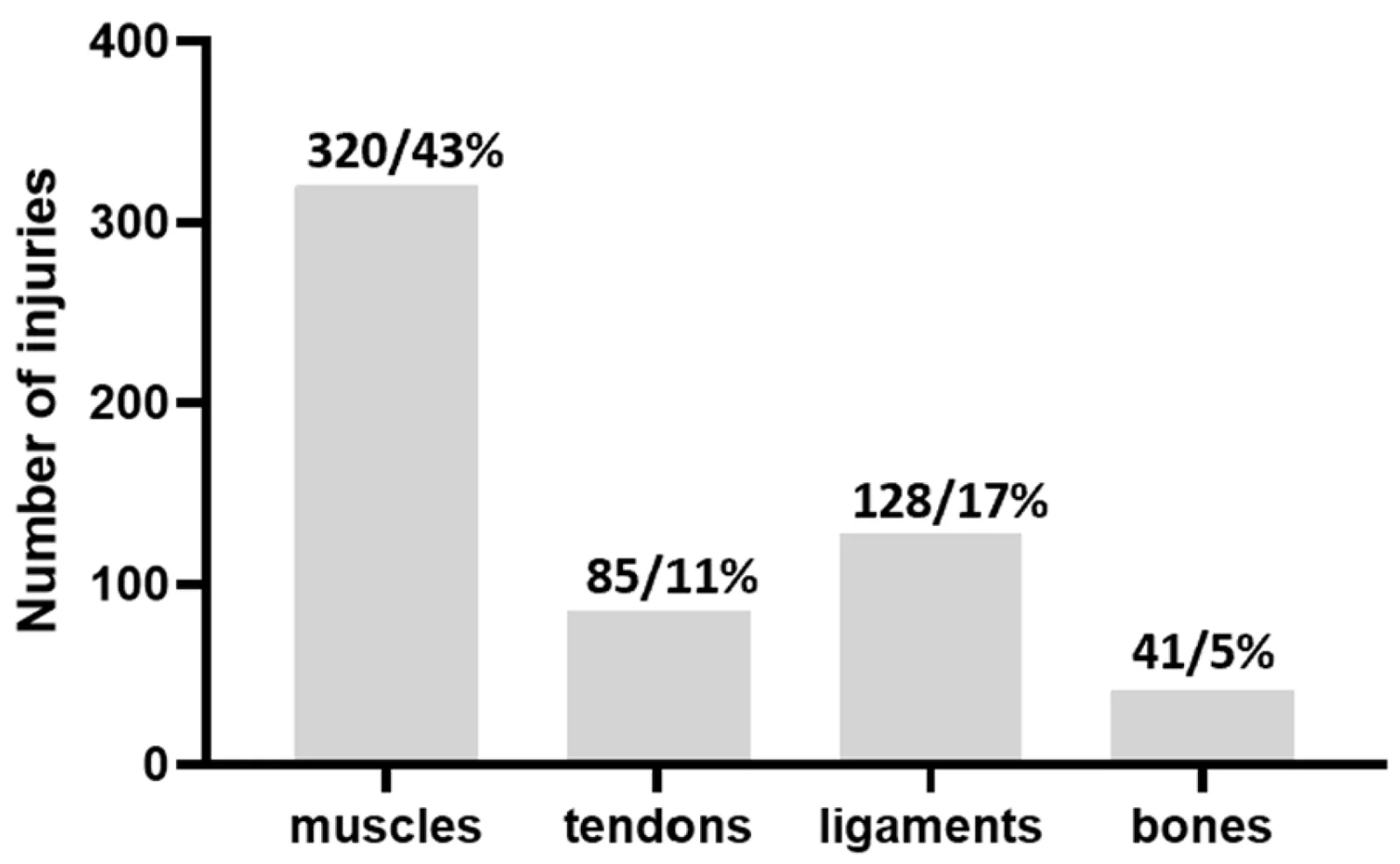
Injuries per body tissue (N/percentage of total injuries).

In terms of injured tissue (Figure 4), muscle injuries predominated (43%), followed by ligament (17%) and tendon injuries (11%). Less frequently affected tissue categories, accounting for 175 injuries, were omitted to improve visual clarity. Percentages were calculated using the 574 injuries represented in the figure and may not sum to exactly 100% because of rounding.

**Figure 5:**
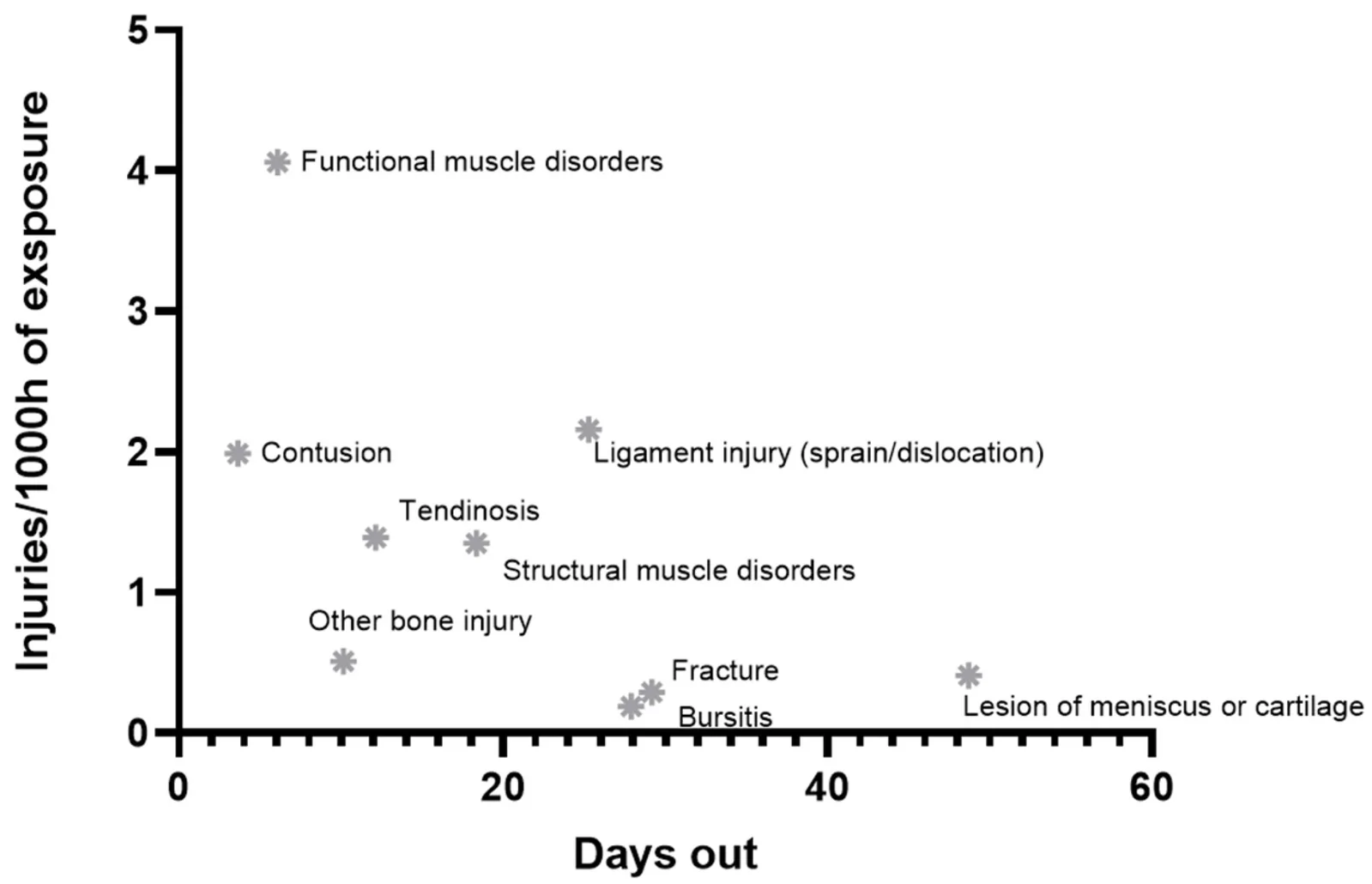
Relative burden of different injury types.

Figure 5 shows the relation between injury incidence and time-loss, illustrating the relative burden of different injury types. Functional muscle disorders were characterised by the highest injury frequency despite a low mean number of days lost, whereas ligament injuries combined a high incidence with a greater mean duration of absence. Meniscal or cartilage lesions and tendon ruptures were associated with the longest recovery times but occurred relatively infrequently.

## 4. Discussion

The aim of this study was to investigate the epidemiology of time-loss injuries in an elite Croatian professional football club over nine consecutive seasons. Several important findings emerged from this study. Overall injury incidence varied across the observation period and averaged 12.66 injuries per 1000 h of exposure, which is slightly higher than that reported in most comparable cohorts. Trend analysis demonstrated a non-significant decline in injury incidence until the COVID-19 season, followed by an increase in the subsequent seasons, although it was also non-significant. Match injury incidence was substantially higher than training injury incidence; muscles were the most frequently affected tissue, and the thigh represented the most commonly injured anatomical region. Regarding injury type, functional muscle disorders (muscle cramps/DOMS) were the most frequently recorded injuries, whereas meniscal and cartilage lesions resulted in the longest average absence from training and competition. Finally, approximately four out of five players sustained at least one time-loss injury during a typical competitive season.

### 4.1. Injury Incidence and Trends

Overall injury incidence averaged 12.66 injuries per 1000 h of exposure, although considerable variation was observed across the nine competitive seasons. This value is slightly higher than that reported in most prospective cohort studies of elite professional football, where overall injury incidence generally ranges between 6 and 10 injuries per 1000 h of exposure [8]. The higher incidence observed in the present study may therefore reflect club-specific factors, seasonal variation, and methodological differences rather than a true increase in injury risk. Direct comparisons between epidemiological studies should be interpreted cautiously. Organisational and sporting factors, including frequent changes in coaching staff, may have contributed to variation in the club’s training environment during the observation period. However, coaching changes were not included as an explanatory variable in the present analysis, and no causal inference can be made. Previous research from the same club demonstrated that injury incidence increased during the first two and four weeks following a coaching change [11]. The authors suggested that modifications in training methodology, workload, and organisational routines immediately after the appointment of a new coach may temporarily elevate injury risk. Given the frequency of coaching changes over the nine-season period (i.e., two coaches per season on average), these transitional phases may have contributed to the overall injury incidence observed herein [23]. Coaching turnover should therefore be considered only as a possible contextual factor rather than an established explanation for the injury incidence observed in the present study.

Injury incidence fluctuated considerably between seasons, but the overall nine-year change was not statistically significant, IRR = 0.85 (95% CI: 0.62–1.16).

On the other hand, the Joinpoint analysis identified two distinct phases, with a non-significant annual decrease in injury incidence of 9.66% from the first season until the COVID-19 season, followed by a non-significant annual increase of 8.75% thereafter. These findings partly agree with the UEFA Elite Club Injury Study, which demonstrated a gradual long-term reduction in injury incidence over an 18-year period without an interruption of the declining trend [8]. A similar change point around the COVID-19 season was reported by Škomrlj et al., who also identified the pandemic period as a turning point in long-term injury trends. Unlike this study, injury incidence in their cohort continued to decline after the pandemic, although at a slower rate than before [24]. The estimated joinpoint corresponded to the 2019/20 season, which coincided with the disruption caused by the COVID-19 pandemic. However, neither the decrease before the joinpoint nor the subsequent increase was statistically significant, and the overall nine-season trend was also non-significant. Therefore, this temporal correspondence should not be interpreted as evidence that the pandemic caused a change in injury incidence. Pandemic-related interruptions, altered competition schedules, reduced preparation periods, and changes in training exposure may have contributed to the observed pattern, but several other contextual factors, including coaching changes, squad turnover, training practices, and medical reporting, could have produced similar seasonal variation. Given the single-club design and the limited number of seasonal observations, these findings should be considered exploratory.

Another important finding was the consistently high proportion of injured players throughout the observation period. Across the complete observation period, 295 of 382 eligible player-seasons included at least one injury, corresponding to a pooled proportion of 77.2% injured player-seasons, with seasonal values ranging from 60% to 98%. In practical terms, this indicates that approximately four out of every five players experienced at least one injury each season. This observation is consistent with previous UEFA data indicating that the vast majority of professional players sustain at least one injury during a competitive season, with a typical squad of 25 players expected to sustain approximately 50 time-loss injuries annually [10]. These observations further emphasise the importance of continuous epidemiological surveillance and the implementation of evidence-based preventive strategies with the ultimate goal of maximising player availability throughout the competitive season [25].

### 4.2. Injury Classification by Type and Tissue

The results in this study showed that muscle-related injuries represented the predominant injury category, irrespective of whether injuries were classified by type or by affected tissue. Functional muscle disorders (muscle cramps/DOMS) were the most frequently recorded injury type, accounting for 240 injuries, followed by ligament injuries and contusions. When classified according to tissue, muscle injuries accounted for almost half of all recorded injuries, with ligament injuries representing the second most affected tissue. These results are consistent with previous epidemiological studies in elite football, which have identified muscle injuries as the predominant injury category, typically accounting for 30–40% of all time-loss injuries, while ligament injuries represent the second most commonly affected tissue [8,12,14]. The high proportion of muscle injuries is not unexpected given the physical demands of professional football, characterised by frequent accelerations, decelerations, sprinting, kicking, and rapid changes in direction. These actions expose the musculature to repeated high mechanical and eccentric loads, particularly during high-speed running and braking actions [3,26].

Functional muscle disorders were the most common injury type, but resulted in relatively short absences from training and competition, with a mean lay-off of 6.1 days. Similarly, contusions, which were predominantly contact-related injuries, caused the shortest average absence (3.6 days). Although meniscal and articular cartilage injuries were relatively uncommon, they resulted in the greatest mean time-loss (48.7 days). This is in accordance with previous studies that indicated intra-articular knee injuries occur relatively infrequently, but with the longest rehabilitation periods in elite football [15]. This distinction illustrates why injury prevention priorities should be based not only on injuries with high frequency but also on injuries with high injury burden, as relatively uncommon injuries may account for a disproportionate loss of player availability.

Structural muscle injuries remained one of the most clinically relevant injury types despite their lower frequency. A total of 80 muscle ruptures (approximately nine per season) were recorded, resulting in a mean absence of 18.4 days, which is comparable with previous reports from elite professional football [27]. The marked differences in lay-off duration between functional and structural muscle injuries reinforce the need to distinguish between these entities during both clinical assessment and injury surveillance. Although functional muscle disorders generally resulted in only short periods of absence, they should not be regarded as clinically insignificant. According to the Munich consensus statement, functional muscle disorders may represent a precursor to structural muscle injury if they are not recognised and managed appropriately [28]. Interestingly, approximately one-third of all functional muscle disorders in the present study were recurrent injuries. This finding may indicate that current management and return-to-play strategies could be further optimised to reduce the risk of recurrence [29]. Clinically, prompt recognition and appropriate management of functional muscle disorders may substantially reduce their overall burden within elite football squads. However, recurrence injuries are multifactorial and may also be influenced by differences in player exposure, individual susceptibility, previous injury history, diagnostic classification, and squad tenure. Further studies incorporating individual-level exposure, rehabilitation characteristics, and return-to-play criteria are needed to clarify the factors associated with these recurrent injuries.

### 4.3. Injuries by Body Parts

The distribution of injuries by anatomical region closely reflects the physical demands of elite football. As expected, the vast majority of injuries involved the lower extremities, with the thigh (201) representing the most frequently affected body region, followed by the knee (125), hip/groin (123), ankle (86), and lower leg (76). Together, these five anatomical regions accounted for more than 80% of all recorded injuries. The predominance of thigh injuries largely reflects the high proportion of muscle injuries observed in the present cohort, reinforcing the close relationship between injury type and anatomical location. This distribution is highly consistent with previous epidemiological studies in elite football. The thigh is consistently recognised as the most commonly injured body region, followed by the knee and ankle [8,10,14,23]. The similarly high number of knee and hip/groin injuries suggests that injury prevention strategies should not focus exclusively on thigh muscle injuries but also address the biomechanical and neuromuscular demands placed on adjacent joints and muscle groups.

The predominance of lower-limb injuries is not surprising given that football performance relies heavily on repetitive high-intensity running, sprinting, kicking, jumping, tackling, and rapid changes in direction, all of which place substantial mechanical demands on the locomotor system [3,26]. Because more than 80% of all injuries involved only five anatomical regions, prevention programmes targeting these areas are likely to have the greatest effect on player availability. However, these findings reflect injury frequency rather than region-specific injury burden. Less frequent injuries, particularly knee injuries, may result in substantially longer absences and a greater overall impact on player availability. Consequently, prevention priorities should not be determined from frequency alone, and future analyses should combine injury incidence with cumulative days lost to quantify burden by anatomical region.

### 4.4. Strengths and Limitations

Several methodological characteristics strengthen the interpretation of the present findings. To our knowledge, this is the first longitudinal epidemiological investigation of injuries in Croatian elite professional football. The nine-season observation period represents one of the longest continuous injury surveillance datasets reported from a single professional club, allowing the assessment of long-term injury trends that would not be detectable in shorter surveillance periods. A further strength is that injury registration was performed prospectively by the same medical staff throughout the entire study period, ensuring methodological consistency in injury diagnosis, classification, and recording. In addition, injuries were systematically classified following FMARC consensus recommendations according to injury type, affected tissue, anatomical location, severity, recurrence, and injury mechanism, allowing for a comprehensive characterisation of the injury profile.

The findings should be interpreted in light of several limitations. First, the findings are based on a single professional football club, which may limit the generalisability of the results to other leagues, countries, or competitive environments. Despite routine medical surveillance, underreporting cannot be excluded, particularly for minor complaints that players did not disclose. The same team physicians remained responsible for diagnosis throughout the observation period; however, continuity of personnel does not eliminate diagnostic variability or potential changes in clinical assessment, imaging use, and injury-management practices over nine seasons. Changes in coaching staff and training practices were also not systematically quantified. These factors may have influenced injury occurrence or recording and could not be disentangled from the observed temporal pattern.

Temporal trends were analysed using aggregated season-level incidence rates. Consequently, the analysis did not explicitly model within-player dependence among players contributing data across multiple seasons or potential overdispersion in individual injury counts. The findings should therefore be interpreted as exploratory club-level temporal patterns rather than individual-level longitudinal associations. Finally, although the principal injury and exposure variables could be aligned with contemporary reporting recommendations, some variables introduced in the more recent IOC and football-specific consensus frameworks were not available for all seasons. Consequently, complete retrospective harmonisation with the updated classification system was not possible.

## 5. Conclusions

This nine-season retrospective study provides a detailed overview of injury epidemiology in elite Croatian professional football. Neither the estimated decline before the 2019/20 joinpoint nor the subsequent increase was statistically significant, and the overall temporal trend was also non-significant. More than 80% of all injuries involved the lower extremities, while muscle injuries accounted for almost half of all recorded injuries. Functional muscle disorders represented the most frequent injury type, whereas meniscal and articular cartilage lesions resulted in the greatest mean time loss per injury.

These findings describe the frequency and consequences of injuries within the observed club and may inform ongoing surveillance and future research. However, anatomical injury frequency alone does not establish burden-based prevention priorities, and the effectiveness of preventive or return-to-play strategies was not evaluated. Further studies incorporating individual and contextual factors are needed to investigate the determinants of injury occurrence and assess targeted interventions.

## Figures and Tables

**Table 1:** Injury incidence.

Season	Overall Injury Incidence, Injuries/1000 h	Training Injury Incidence, Injuries/1000 h	Match Injury Incidence, Injuries/1000 h	IRR 1–9 Years (95% CI)
**15/16**	12.80 (10.25–15.78)	9.91 (7.56–12.75)	36.36 (23.96–52.91)	0.85 (0.62–1.16)
**16/17**	17.93 (14.87–21.45)	12.78 (10.07–15.99)	59.26 (43.06–79.55)	
**17/18**	14.26 (11.57–17.40)	6.97 (5.02–9.42)	70.92 (53.43–92.31)	
**18/19**	11.53 (9.10–14.41)	6.70 (4.79–9.13)	52.15 (36.72–71.88)	
**19/20**	9.21 (7.16–11.65)	5.41 (3.81–7.46)	48.48 (33.16–68.45)	
**20/21**	8.04 (5.79–10.86)	3.98 (2.36–6.30)	33.83 (21.67–50.33)	
**21/22**	15.64 (12.69–19.06)	9.18 (6.83–12.06)	66.24 (48.67–88.09)	
**22/23**	13.18 (10.56–16.26)	9.67 (7.30–12.56)	38.34 (26.05–54.42)	
**23/24**	10.88 (8.51–13.70)	6.62 (4.71–9.05)	45.45 (31.29–63.84)	
**ALL**	12.66 (11.77–13.60)	7.97 (7.22–8.77)	50.13 (44.86–55.84)	

LEGEND—IRR -Incidence rate ratio between season 1 and 9.

**Table 2:** Injured players, injury burden and total injuries per season.

Season	Eligible Player-Seasons	Injured Players-Season	Proportion of Injured Player-Seasons	Total Injuries	Days/1000 h
**15/16**	39	32	82%	87	151.64
**16/17**	42	41	98%	120	87.43
**17/18**	38	31	82%	97	127.93
**18/19**	40	32	80%	77	96.61
**19/20**	45	31	69%	69	41.64
**20/21**	40	24	60%	42	39.99
**21/22**	45	35	78%	98	130.51
**22/23**	49	37	76%	87	75.76
**23/24**	44	32	73%	72	260.41
**TOTAL**	382	295	77%	749	113.11

**Table 3:** Descriptive data of injuries by injury type.

Injury Type	Days Out	Number of Injuries	Recurrent Injury	Overuse	Trauma	Unknown Mechanism	Match	Training	Contact	Non-Contact
**Functional muscle disorders** **Muscle cramps (DOMS)**	6.1	240	91	235	4	1	81	159	2	238
**Ligament injury (sprain/dislocation)**	25.28	128	26	9	106	13	61	67	47	81
**Contusion**	3.63	118	8	0	118	0	76	42	111	7
**Tendinosis**	12.14	82	24	68	11	3	26	56	3	79
**Structural muscle disorders** **Muscle rupture**	18.36	80	19	5	70	5	46	34	10	70
**Other bone injury**	10.16	30	19	22	8	0	13	17	4	26
**Lesion of the meniscus or cartilage**	48.7	24	11	8	13	3	8	16	3	21
**Bursitis**	29.18	17	0	5	8	4	5	12	5	12
**Fracture**	27.9	11	0	1	9	1	6	5	9	2
**Laceration**	3	5	0	0	5	0	2	3	4	1
**Concussion**	20.75	5	0	0	4	1	4	1	4	1
**Nerve injury**	14	3	0	2	1	0	1	2	0	3
**Tendon rupture**	48	3	0	0	2	1	2	1	2	1

## Data Availability

The data analysed in this study are not publicly available because they originate from confidential medical records of players from a single professional football club and may carry a risk of indirect identification. De-identified aggregate data may be made available by the corresponding author upon reasonable request and subject to ethical and club approval.
